# Antibacterial Biomaterial Based on Bioglass Modified with Copper for Implants Coating

**DOI:** 10.3390/jfb14070369

**Published:** 2023-07-13

**Authors:** Imen Hammami, Sílvia Rodrigues Gavinho, Suresh Kumar Jakka, Manuel Almeida Valente, Manuel Pedro Fernandes Graça, Ana Sofia Pádua, Jorge Carvalho Silva, Isabel Sá-Nogueira, João Paulo Borges

**Affiliations:** 1 I3N and Physics Department, Aveiro University, 3810-193 Aveiro, Portugal; imenhammami@ua.pt (I.H.); silviagavinho@ua.pt (S.R.G.); suresh@ua.pt (S.K.J.); mav@ua.pt (M.A.V.); mpfg@ua.pt (M.P.F.G.); 2 I3N-CENIMAT and Physics Department, NOVA School of Science and Technology, Campus de Caparica, 2829-516 Caparica, Portugal; as.padua@campus.fct.unl.pt (A.S.P.); jcs@fct.unl.pt (J.C.S.); 3 Associate Laboratory i4HB—Institute for Health and Bioeconomy, NOVA School of Science and Technology, NOVA University Lisbon, 2819-516 Caparica, Portugal; isn@fct.unl.pt; 4 UCIBIO—Applied Molecular Biosciences Unit, Department of Life Sciences, NOVA School of Science and Technology, NOVA University Lisbon, 2819-516 Caparica, Portugal; 5 I3N-CENIMAT and Materials Science Department, NOVA School of Science and Technology, Campus de Caparica, 2829-516 Caparica, Portugal

**Keywords:** Bioglass^®^, copper, antibacterial activity, bioactivity, osseointegration, implant coating

## Abstract

Biofilm-related implant infections pose a substantial threat to patients, leading to inflammation in the surrounding tissue, and often resulting in implant loss and the necessity for additional surgeries. Overcoming this implantology challenge is crucial to ensure the success and durability of implants. This study shows the development of antibacterial materials for implant coatings by incorporating copper into 45S5 Bioglass^®^. By combining the regenerative properties of Bioglass^®^ with the antimicrobial effects of copper, this material has the potential to prevent infections, enhance osseointegration and improve the long-term success of implants. Bioglasses modified with various concentrations of CuO (from 0 to 8 mol%) were prepared with the melt-quenching technique. Structural analysis using Raman and FTIR spectroscopies did not reveal significant alterations in the bioglasses structure with the addition of Cu. The antibacterial activity of the samples was assessed against *Gram-positive* and *Gram-negative* bacteria, and the results demonstrated significant inhibition of bacterial growth for the bioglass with 0.5 mol% of CuO. Cell viability studies indicated that the samples modified with up to 4 mol% of CuO maintained good cytocompatibility with the Saos-2 cell line at extract concentrations up to 25 mg/mL. Furthermore, the bioactivity assessment demonstrated the formation of a calcium phosphate (CaP)-rich layer on the surfaces of all bioglasses within 24 h. Our findings show that the inclusion of copper in the bioglass offers a significant enhancement in its potential as a coating material for implants, resulting in notable advancements in both antibacterial efficacy and osteointegration properties.

## 1. Introduction

The use of implantable medical devices, such as orthopedic or dental implants, has become common practice in almost all fields of medicine. However, foreign bodies are associated with a significant risk of bacterial infections. These infections are a significant concern in healthcare settings, and can lead to serious complications. The biofilms, which are communities of bacteria encased in a protective matrix, can form on the surface of the implants, and trigger an inflammatory response in the surrounding tissue, leading to further complications. In the case of dental implants, biofilm formation plays a significant role in the development of peri-implantitis, which is a chronic inflammatory disease caused by anaerobic *Gram-positive* and *Gram-negative* bacteria that gradually leads to bone loss and implant failure [1,2,3,4]. Despite taking all necessary precautions, such as maintaining patient asepsis and ensuring sterilization of instruments, infections can still occur after surgery. The treatment of implant-related infections typically involves a combination of approaches, with antibiotics being commonly employed. However, their effectiveness against biofilm-associated infections is limited [5,6]. The protective matrix of biofilms can prevent antibiotics from reaching the bacteria, making them less effective. Consequently, addressing biofilm-associated infections may necessitate surgical intervention to remove the infected device, which places an additional burden on the patient and increases the associated surgical risks. Prioritizing preventive solutions to prevent bacterial colonization during surgery is crucial for minimizing the risk of implant-related infections. One effective approach is the development of antibacterial materials for implant coatings that are capable of directly combating bacteria at the infection source [7,8,9]. Moreover, the surface modification of the implant enhances material–bone interaction and, therefore, stability [10,11]. Several studies have demonstrated the impact of implant surface roughness on cell integration and its association with increased osteointegration at the clinical level [12,13,14]. Increased surface roughness has been found to significantly affect cell behavior, leading to a higher expression of integrin for osteoblastic cells. This promotes osteoblast differentiation and cell proliferation [15].

The 45S5 bioglass^®^ (46.1% SiO_2_, 24.4% Na_2_O, 26.9% CaO and 2.6% P_2_O_5_ (mol%)), developed by Hench et al., has emerged as a highly successful material for implant coating due to its distinctive properties [16,17]. The release of antimicrobial ions, notably sodium and calcium, from the bioglass coating disrupts the cell membranes of bacteria and fungi, impeding their proliferation. Furthermore, the bioactive nature of bioglass facilitates the formation of a hydroxyapatite layer upon contact with bodily fluids, promoting osseointegration and stimulating new bone growth. By enhancing stability and integration with surrounding tissues, the bioglass coating effectively deters bacterial colonization on implant surfaces [17,18,19,20].

In recent years, efforts have been undertaken to enhance the biological performance of bioactive glasses by integrating metallic ions into the glass network [4,21,22,23,24,25,26]. Copper (Cu), in particular, has garnered significant attention due to its potential antibacterial effects when released in the physiological environment upon the dissolution of the bioglass matrix. The presence of Cu ions leads to the generation of reactive oxygen species (ROS), which can cause oxidative stress and damage cellular components in bacteria [27,28,29]. Additionally, Cu can induce lipid peroxidation, disrupting the integrity of bacterial cell membranes. The oxidation of proteins and DNA by Cu ions further impairs bacterial functions and viability [27,28,29]. In the case of human cells, they have sophisticated antioxidant defense systems that neutralize excess ROS to maintain cellular homeostasis. These defense mechanisms include enzymes such as superoxide dismutase, catalase and glutathione peroxidase, which detoxify ROS and protect the cells from oxidative damage [30,31]. The combined action of these processes contributes to the antimicrobial activity exhibited by copper ions, making them effective agents for incorporation into the bioglass matrix. Additionally, copper plays a significant role in the regulation of angiogenesis by promoting endothelial cell proliferation and migration [32,33]. Moreover, studies have demonstrated that Cu can induce an increase in the differentiation of mesenchymal stem cells (MSCs) into osteoblasts, promoting bone formation and mineralization [34,35]. Although copper is recognized for its pivotal role in hemostasis and bone formation, an excess amount of this element can be cytotoxic [36]. Studies have reported that the body tolerates low doses of copper, typically up to 8.66 mg kg^−1^, while doses ranging from 50 to 54.4 mg kg^−1^ can be lethal due to the generation of free radicals, resulting in toxicity and inflammatory effects [36,37,38].

The present study focused on the synthesis of copper-modified 45S5 bioactive glass through the melt-quenching method, to explore its potential as a coating material for dental implants. The synthesized bioglasses with varying percentages of copper oxide (from 0 to 8 mol%) were subjected to comprehensive morphological, structural and biological investigations. This research encompasses a combination of these specific analyses that collectively contribute to a comprehensive understanding of the biological properties of the copper-modified bioglass. By examining the physicochemical properties, we have gained insights into the material’s composition and structural characteristics, which can impact its biocompatibility and bioactivity. Additionally, we have assessed the antibacterial activity, demonstrating the potential of the copper modification to confer enhanced antimicrobial properties to the bioglass. The cytotoxicity assessment has provided valuable information on the material’s compatibility with the human osteosarcoma cell line (Saos-2 cells), while the bioactivity analysis aimed to determine its ability to promote bone integration and regeneration. The findings of this study provide valuable insights into the development of advanced biomaterials for dental implant applications.

## 2. Materials and Methods

### 2.1. Synthesis Method

A series of 45S5 bioglass^®^ samples (46.1 SiO_2_, 24.4 Na_2_O, 26.9 CaO, 2.6 P_2_O_5_, mol%) incorporating varying percentages of copper oxide (CuO), from 0 to 8 mol% (named Cu0, Cu0.25,…Cu8), were successfully synthesized using the melt-quenching technique. The chemical precursors, including SiO_2_, P_2_O_5_, CaCO_3_, Na_2_CO_3_ and CuO, with a high purity grade (>99.99%), were initially thoroughly mixed using a planetary ball milling process for 1 h at 300 rpm. The resulting mixed powder was calcined at 800 °C for 8 h. The calcined powder was then carefully melted, using a platinum crucible, at 1300 °C for 1 h, ensuring regular hand mixing to improve the melt homogeneity. After the quench, the resulting bulk glass samples were then finely ground and milled into powder form using the same planetary ball milling process, for 1 h at 500 rpm.

### 2.2. Structural and Physicochemical Characterization

FTIR spectra in the range of 400–1200 cm^−1^ were collected using the FT Perkin-Elmer Spectrum BX Spectrometer (Waltham, MA, USA) in the ATR crystal (Golden Gate Diamond ATR accessory). The measurements were performed using powder samples. Throughout the acquisition, the room temperature and humidity were maintained at approximately 23 °C and 35%, respectively, to ensure consistent conditions.

Raman spectroscopy was performed at room temperature utilizing a Horiba Jobin Yvon HR 800 spectrometer (Longjumeau, France) equipped with an Ar + laser (λ = 532 nm). The measurements were conducted employing a back-scattering geometry across the spectral range of 200 to 1500 cm^−1^. A 50× lens was employed to precisely focus on the sample during data collection.

Photoluminescence (PL) emission spectra were obtained on a Horiba Jobin Yvon Fluorolog-3 instrument equipped with a continuous Xe lamp of 450 W and photomultiplier (PMT) detector, in the range of 400–700 nm with a step width of 0.5 nm with the source, sample and the detector placed in orthogonal geometry.

### 2.3. Morphological Characterization

The surface morphology of the glass samples pellets produced from the synthesized powders using a uniaxial pressure system and cylindrical steel mold, was assessed using a TESCAN Vega 3 scanning electron microscope (SEM), Brno, Czech Republic. The chemical composition of the samples was semi-quantitatively analyzed using the Bruker QUANTAX EDS (energy dispersive spectroscopy) system coupled to the Vega 3 SEM. A 5 µm diameter electron beam spot was utilized to examine specific surface sites on each sample.

### 2.4. Cytotoxicity Assay

The cytotoxicity assessment of the samples followed the extract method, and utilized the human osteosarcoma cell line (Saos-2 cells, ATCC^®^ HTB-85™) in compliance with the International Standard ISO 10993-5. Prior to the evaluation, the bioglass powders underwent sterilization at 120 °C for 2 h. Two types of extract, non-passivated and passivated, were generated by exposing the samples to culture medium (McCoy 5A medium, from Merck KGaA, Darmstadt, Germany, supplemented with 10% fetal bovine serum, from Biowest, France, and 1% penicillin, 100 U/mL, and streptomycin, 100 µg/mL, Gibco, ThermoFisher, Waltham, MA, USA) at a concentration of 100 mg/mL. For the non-passivated extract, the powder in contact with the medium was incubated for 24 h at 37 °C, filtered through a 0.22 µm cellulose acetate filter, and stored at 37 °C. In the case of the passivated extract, fresh McCoy 5A medium was added to the same bioglass powder and incubated for 24 h at 37 °C.

Saos-2 cells were seeded onto 96-well plates at a density of 30 k cells per cm^2^ and incubated for 24 h at 37 °C with a 5% CO_2_ atmosphere. Subsequently, the culture medium was replaced by the non-passivated and passivated extracts. In addition to the initial concentration of 100 mg/mL, four serial dilutions were prepared (50 mg/mL, 25 mg/mL, 12.5 mg/mL and 6.25 mg/mL). A positive control (cells in a cytotoxic environment, caused by the supplementation of the culture medium with 10% dimethyl sulfoxide) and a negative control (cells cultured with normal medium) were set up. After 48 h of incubation, the cell viability was assessed using the resazurin cell viability indicator. The optical absorbances of each well were measured at 570 nm and 600 nm using a Biotek ELX800 microplate reader. To ensure the reproducibility of the results, the study was performed in triplicate with six replicates in each experiment.

### 2.5. Antibacterial Activity

The antibacterial potential of bioactive glasses with varying copper concentrations was investigated against the reference strains *Escherichia coli* K12 DSM498 (DSMZ, Braunschweig, Germany), *Staphylococcus aureus* COL MRSA (methicillin-resistant strain, obtained from Rockefeller University) and *Streptococcus mutans* DSM20523 (DSMZ, Braunschweig, Germany). All of the microorganisms were cultured at 37 °C in tryptic soy broth (TSB) medium and *S. mutans* in a 5% CO_2_ incubator. Before conducting the experiments, bioglass powder pellets measuring 6 mm in diameter and approximately 2 mm in thickness were subjected to sterilization at 180 °C for 2 h.

The agar diffusion assay using the two-layer bioassay method was employed, involving the use of TSB medium solidified with agar 1.5% *w*/*v* for the base layer, and with agar 0.8% *w*/*v* for the top layer. Preparation of the assay plates entailed pouring 18–20 mL of the base layer, followed by the addition of 4 mL of molten seeded overlay containing approximately 10^7^–10^8^ CFU/mL of the respective indicator bacteria grown overnight, as described above. The pellets were positioned in the center of the plate, left at room temperature for 4 h and subsequently, the plates were incubated at 37 °C for 24 h.

Images of the pellets were taken, and the diameter of the resulting inhibition zone was determined using ImageJ software. To ensure accuracy, each pellet underwent meticulous measurement from multiple orientations, with a total of 30 measurements per pellet. Statistical analysis of the data was performed using GraphPad Prism 8.0 software, employing an unpaired *t*-test, to compare the antibacterial effects of the bioactive glass base composition with each of the different samples.

### 2.6. In Vitro Bioactivity Assay

Following the ISO 23317:2017 standard, the evaluation of bioactivity for the bi-oglasses samples was conducted by transforming the powders in disk form (pellets) with a diameter of 7 mm, and then immersing them in a simulated bodily fluid (SBF). The pellets, placed in various flasks and immersed in SBF, were then incubated at a constant temperature of 37 °C for different times (12, 24, 48, 96 h, 14 and 28 d). Throughout the incubation period, the samples were placed on continuous oscillating support to mimic the continuous flow of biological fluids. The SBF solutions were refreshed every 48 h to simulate the biological environment.

The calculation of the SBF volume required for each sample followed a specific formula:

V_s_ = 100 mm × S_a_, (1)

where V_s_ is the volume of SBF in mm^3^, and S_a_ is the surface area of the pellet in mm^2^.

Once the immersion period concluded, the pellets were taken out from the SBF medium, gently cleansed using deionized water, and allowed to dry at room temperature. Subsequently, the samples were subjected to SEM/EDS analysis. This analysis aimed to determine the change in ion concentration and the development of an apatite-like layer on the surface over 28 d.

## 3. Results and Discussion

### 3.1. Structural and Physicochemical Characterization

**Figure 1:**
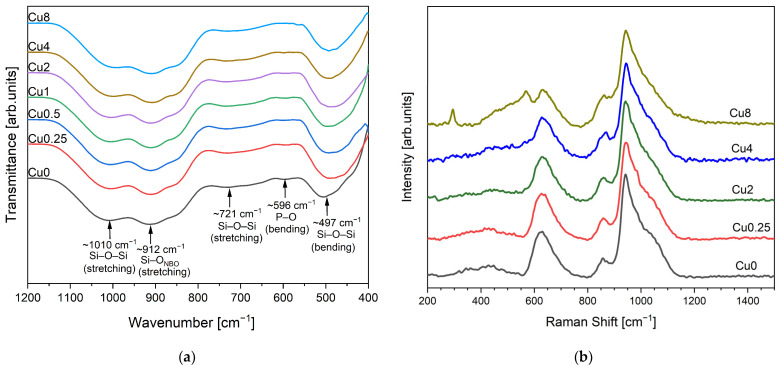
(**a**) FTIR spectra and (**b**) Raman spectra of bioactive glasses modified with CuO.

The FTIR spectra of the glasses revealed the following features, as shown in Figure 1a. The bands observed at around 1010 cm^−1^ and 721 cm^−1^ were assigned to Si–O–Si stretching modes [22,35,39,40,41,42,43]. The appearance of a band at 912 cm^−1^, assigned to Si–O_NBO_ stretching mode [22,35,39,40,41,42,43], demonstrates the existence of the non-bridging oxygen ions. The band located at 596 cm^−1^ is related to a P-O-P bending mode [22,35,40,41,42,43,44]. The presence of a band at 497 cm^−1^ is associated with an Si-O-Si bending mode [22,35,39,40,41,42,43]. The observed shift of this band towards lower wavenumbers with the increase in copper content is caused by the presence of copper, which leads to a change in the degree of polymerization of the glass structure. The FTIR measurements do not show any type of modification with the insertion of copper ions in the glass matrix.

**Figure 2:**
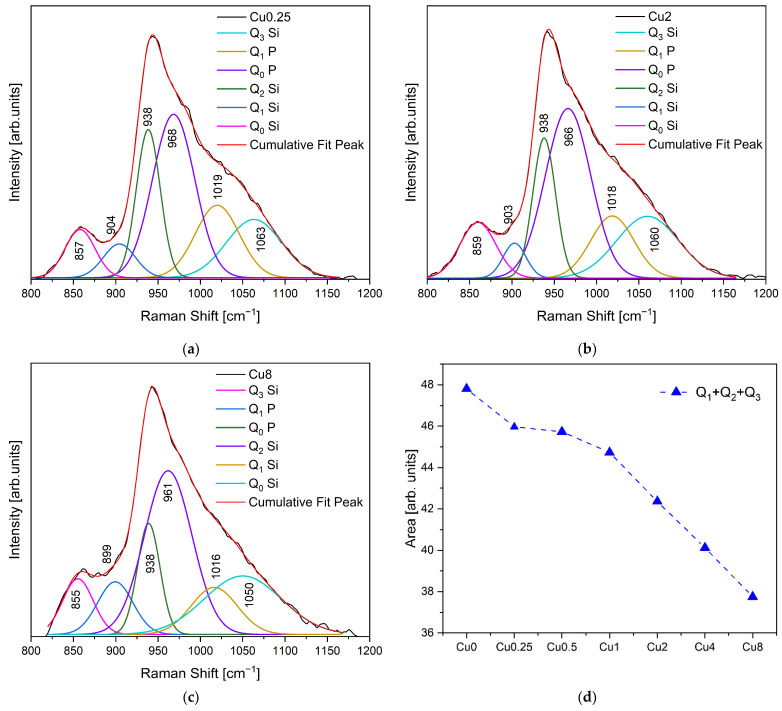
Deconvolution of Raman spectra for the (**a**) Cu0.25, (**b**) Cu2 and (**c**) Cu8 samples (R^2^ > 0.999); and (**d**) sum of areas of Q_0_ and Q_1_ + Q_2_ + Q_3_ (NBOs) units of the different bioglass samples.

The Raman spectra for the modified bioglasses by CuO are displayed in Figure 1b, revealing a comparable trend among them. Nevertheless, with increasing the Cu concentration to 8 mol%, two distinct bands corresponding to the Ag and Bg modes of CuO become evident at 293 and 569 cm^−1^, respectively [45,46]. The vibrational modes of asymmetric and symmetric stretching in the high-frequency region (800 and 1200 cm^−1^) are considered particularly significant for silicate glasses. Figure 2 depicts the deconvolution of Raman spectra in this region for the Cu0.25, Cu2 and Cu8 samples. Six discernible vibrational modes can be identified at 855–859 cm^–1^, 899–904 cm^−1^, 938cm^−1^, 961–968 cm^−1^, 1016–1019 cm^−1^ and 1050–1063 cm^−1^, which are associated with Q_0_ Si units Q_1_ Si, Q_2_ Si, Q_0_ P, and Q_1_ P units and Q_3_ Si units, respectively [22,44,47,48].

Figure 2d illustrates the variation in the sum of the area of Raman vibration bands Q_1_, Q_2_ and Q_3_ units associated with non-bridging oxygen ions (NBOs) as a function of Cu concentration. The results show that the NBO amount decreases with the rise in copper concentration, suggesting an increase in the connectivity of the glass network. The samples Cu0.25 and Cu0.5 exhibited a similar concentration of NBOs.

**Figure 3:**
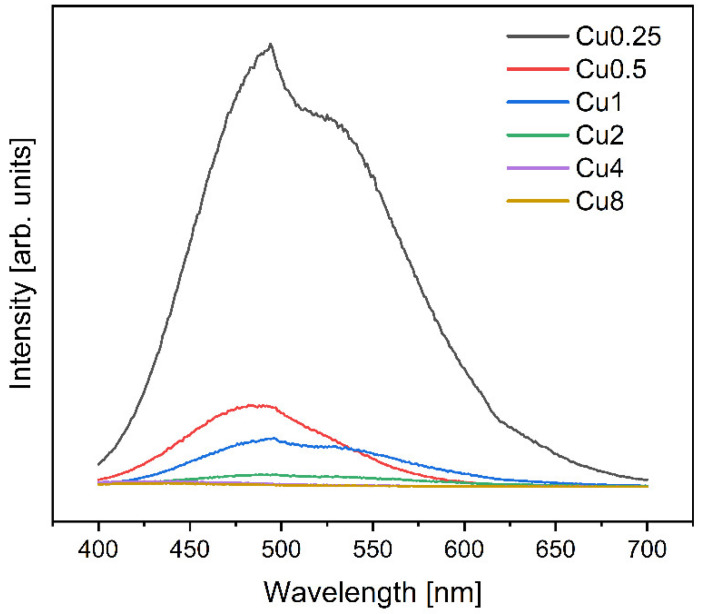
PL emission spectra excited at 280 nm of the bioglasses modified with copper.

Figure 3 displays the photoluminescence (PL) spectra of the glass samples modified with copper upon excitation at 280 nm. Ultraviolet excitation induces significant and wide emission peaks in the visible range in copper-containing glasses, and it is worth noting that copper can be introduced into the glass network in two different oxidation states, Cu^2+^ and Cu^+^ [49,50]. The excitation bands of these ions are located in the UV region [51,52]. The spectral analysis revealed the presence of one main peak at around 475 nm, accompanied by a shoulder at 523 nm. The luminescence peak at 475 nm is associated with the 3d^9^4s^1^–4s^2^3d^10^ transition of Cu^2+^ ions [51,53]. The shoulder peak at 523 nm is a consequence of the transition from the degraded T_1g_ and T_2g_ levels to the ^1^A_g_ energy level of the Cu^+^ copper ions [51,54]. This transition arises from the interaction between NBOs with the Cu^2+^. Therefore, the PL spectra indicate the existence of both Cu^+^ and Cu^2+^ ions, contributing to the observed emission peaks. It was also observed that with the increase in CuO concentration, the PL intensity decreased; this is attributed to the loss of excited energy from copper ions to the host lattice [51]. The decrease in the PL intensity with higher concentration made the PL signal of the Cu8 sample indistinguishable from the noise.

**Figure 4:**
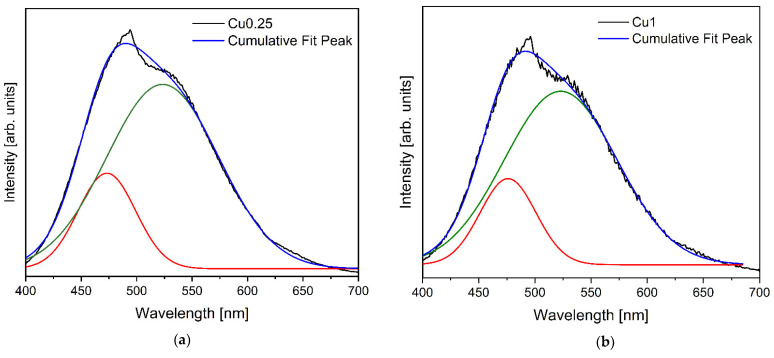

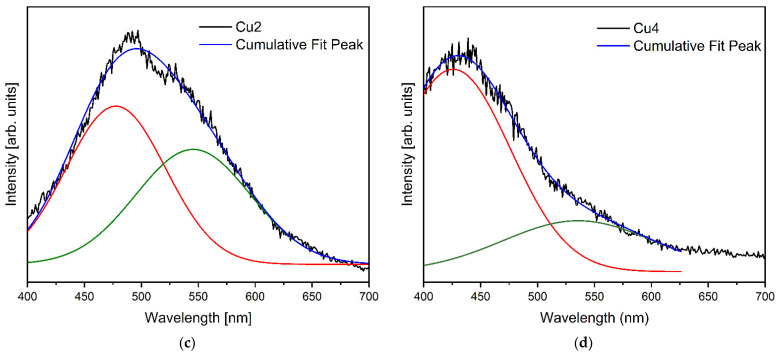
Deconvolution of the PL spectrum of the (**a**) Cu0.25, (**b**) Cu1, (**c**) Cu2 and (**d**) Cu4 bioglass samples.

Figure 4 depicts the deconvolution of the PL spectrum for bioglasses with different CuO content. When the spectra were normalized, the relative intensities of both peaks were changing in such a way that the intensity of the peak at 475 nm due to Cu^2+^ ions increased with respect to the Cu^+^ peak at 523 nm with the rise in Cu concentration inserted into the bioglass. This observation is correlated with the results obtained with the Raman analysis, which shows the decrease in NBOs with the increase in copper concentration inserted into the bioglass (Figure 2d) due to the increase in Cu^2+^ ions at the expense of Cu^+^, thus forming stronger bonds with oxygen and leading to a decrease in the number of available NBOs.

### 3.2. Morphological Characterization

**Figure 5:**
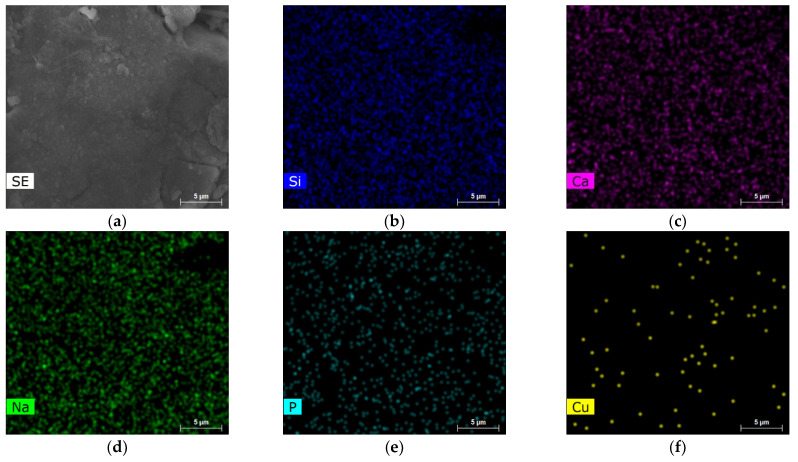
SEM/EDX analyses of Cu0.25 sample (**a**) SEM image, EDS elemental mapping of (**b**) Si, (**c**) Ca, (**d**) Na, (**e**) P and (**f**) Cu.

**Figure 6:**
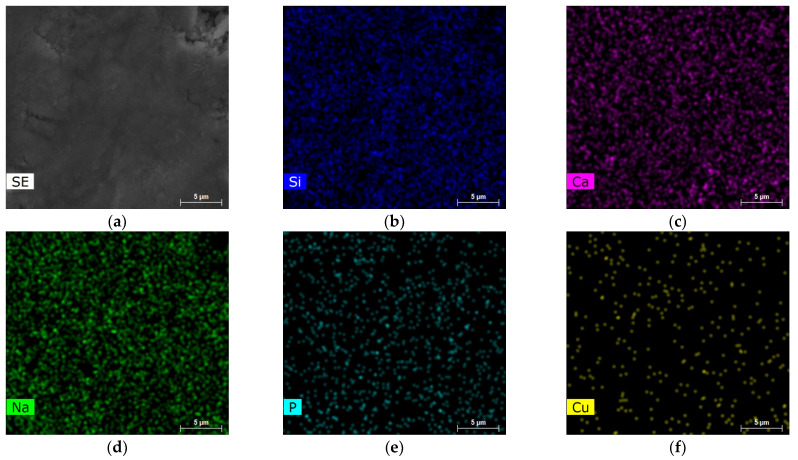
SEM/EDX analyses of Cu8 sample (**a**) SEM image, EDS elemental mapping of (**b**) Si, (**c**) Ca, (**d**) Na, (**e**) P and (**f**) Cu.

SEM–EDS elemental mapping of the Cu0.5 and Cu8 samples are reported in Figure 5 and Figure 6, respectively. The results obtained show the homogenous distribution of the Si, Ca, Na, P and Cu elements.

### 3.3. Cytotoxicity Assay

**Figure 7:**
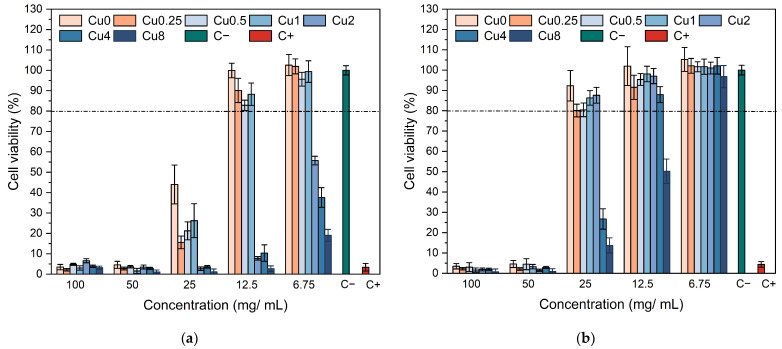
Relative viabilities of (**a**) non-passivated and (**b**) passivated bioglass extracts modified by CuO in culture with Saos-2 cells.

In order to explore the biocompatibility of various bioglass compositions for their potential in bone regeneration, Saos-2 cell line viability was evaluated upon exposure to bioglass extracts. Employing a resazurin assay as an indicator of cell viability, the impact of extract–cell line interactions was examined. The results demonstrate that non-passivated extracts, i.e., those not preconditioned with McCoy’s culture medium, induced a drastic decline in cell viability at concentrations of 100 mg/mL and 50 mg/mL, thus exhibiting a potent cytotoxic effect. When the extract concentration was reduced to 25 mg/mL, the samples with a Cu content lower than 2 mol% demonstrated enhanced cell viability, despite the discernible presence of their cytotoxic attributes. However, when the extract was diluted to 12.5 mg/mL, a noticeable reduction in cytotoxicity was observed for the samples modified with low concentrations of copper, exhibiting a significantly improved cell viability compared to the sample containing copper concentrations of 2 mol% and above. These findings strongly indicate that the introduction of CuO into the bioactive glass confers diminished biocompatibility to the materials, which aligns with the findings of prior research studies [55,56]. By subjecting the materials to a passivation process, as depicted in Figure 7b, the cytotoxicity of the extracts can be alleviated. It is important to note that cytotoxicity is linked to a rise in local pH caused by ion-exchange reactions upon contact of the sample with the cell culture medium within the initial 24 h [42]. Upon interaction with the cellular medium, bioactive glass experiences the breakdown of its Si-O-Si bonds, leading to the release of soluble silica in the form of Si(OH)_4_ into the solution. Consequently, the dissolution rate and pH of the surrounding environment are elevated, thereby influencing cellular metabolism and function. However, when the samples are passivated, the influence of pH alkalinization caused by bioactive glasses diminishes. The passivated extracts derived from the bioglasses modified with copper from 0 to 2 mol% demonstrated a remarkable lack of cytotoxicity at a concentration of 25 mg/mL, whereas the samples Cu4 and Cu8 exhibited no cytotoxic effects at extract concentrations of 12.5 mg/mL and 6.75 mg/mL, respectively. These findings indicate that at these particular concentrations, the samples no longer present a risk to the organism since they can be effectively regulated through natural in vivo pH regulatory mechanisms [57,58].

### 3.4. Antibacterial Activity

In the realm of implantation therapy, tackling bacterial infections has become a crucial aspect. While 45S5 bioactive glass has shown exceptional properties in promoting bone regeneration and inhibiting bacterial growth [59,60,61], it is crucial to explore the potential of bioglass modified with copper in exerting antibacterial effects.

**Figure 8:**
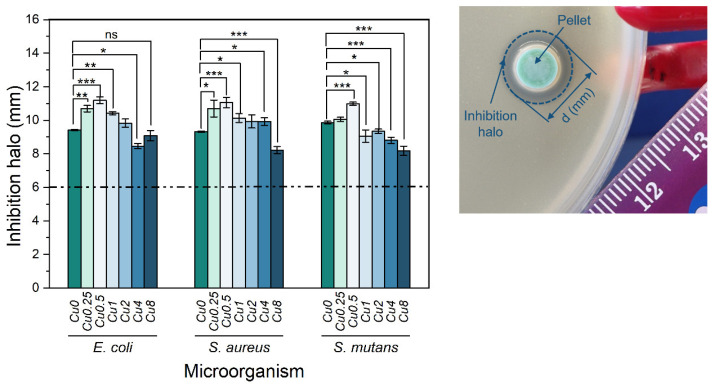
Measurements of inhibition halo diameters of the bioglasses modified with Cu against *E. coli*, *S. aureus* and *S. mutans* bacteria after incubation for 24 h. Results are reported as mean ± SD. The asterisks indicate significance in an unpaired *t*-test; * *p* ≤ 0.05; ** *p* ≤ 0.01; *** *p* ≤ 0.001; ns: non-significant. The image on the right side is an example of an essay plate illustrating the inhibition halo of a Cu0.5 pellet on *E. coli*.

The results presented in Figure 8 illustrate the outcomes of the antibacterial properties assessment conducted on the bioglasses using the agar disc diffusion method. The findings substantiate the antibacterial activity exhibited by all the samples, as evidenced by the presence of inhibition zones surrounding the bioglass pellets. These zones exhibited mean values exceeding 6 mm, corresponding to the diameter of the pellets. The antibacterial effect of 45S5 bioglass against specific bacteria can be attributed to two principle mechanisms: the pH changes towards alkalinity, and the osmotic pressure resulting from the release of bioglass ions, notably Na^+^ and Ca^2+^, into the surrounding medium [59,62]. The alkaline pH range is detrimental to bacterial growth and metabolic activities, resulting in the disruption of proteins and enzymes, and the inhibition of their normal functions. Furthermore, the release of ions and subsequent variations in their concentrations within the bacterial environment impact the integrity of the bacterial cell membrane and intramembrane pressure. Consequently, these alterations evoke modifications in cellular dimensions, morphology, as well as membrane tension levels, ultimately leading to bacterial death. Moreover, several studies showed the potential antibacterial effect of copper due to its capacity to stimulate the production of ROS, which induces oxidative stress and damage to cellular components [63,64,65,66]. Additionally, copper ions directly interact with bacterial DNA, causing DNA damage and genetic instability. According to the results, the Cu0.5 sample exhibited the most potent antimicrobial activity among all the Cu-modified bioglass samples. It demonstrated mean inhibition halo sizes of 11.19 mm, 11.05 mm and 10.98 mm against *E. coli*, *S. aureus* and *S. mutans* bacteria, respectively. However, when the Cu concentration surpassed 0.5 mol%, a noticeable reduction in the inhibition halo size was observed. This reduction indicates a simultaneous decline in the antibacterial effectiveness of the bioglass. In fact, the insertion of Cu into the glass network led to the decrease in NBOs concentration, as shown in Figure 2d. The samples containing the highest percentages of copper showed a reduction in NBOs compared to the bioglass base. The decrease in NBO numbers suggests a reduction in ions released, such as Na^+^ and Ca^2+^, which can impact the antibacterial activity with increasing copper concentration. Moreover, the presence of different oxidation states of copper can affect the antibacterial properties of the bioglass. Previous studies showed that Cu^+^ ions exhibited significantly more antibacterial effects than Cu^2+^ ions [67,68,69]. Therefore, the decrease in the antibacterial effect for the glass with a high concentration of Cu may be associated with increased concentrations of Cu^2+^ compared to Cu^+^, as shown in Figure 4.

### 3.5. In Vitro Bioactivity Assay

**Figure 9:**
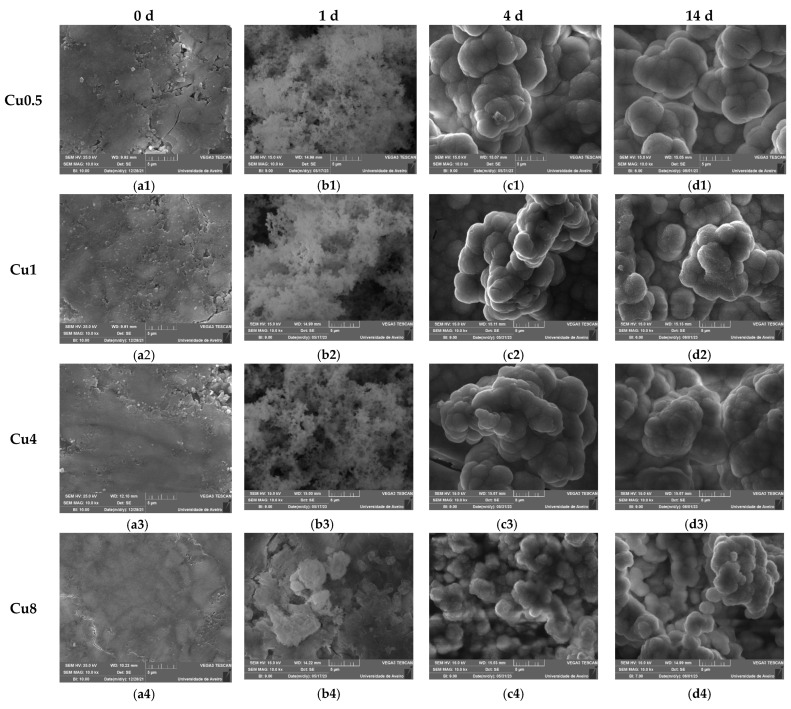
SEM micrographs of bioglass pellets modified with various concentrations of Cu after immersion in SBF for (**a1**–**a4**) 0 d; (**b1**–**b4**) 1 d; (**c1–c4**) 4 d; (**d1**–**d4**) 14 d. (The magnification of the SEM images is 10 kX).

For a comprehensive evaluation of the in vitro bioactivity of synthesized bioglasses within a biological environment, we employed the SBF immersion test as a reliable methodology. This test enables a thorough exploration of the physicochemical interactions between bioactive glass and physiological fluids. Surface chemistry modifications, particularly the development of an apatitic layer, play a vital role in assessing bioglass in vitro, as it profoundly influences the proliferation and adhesion of osteoblast cells [70]. Micrographs acquired via SEM at the surface of bioglass samples after 0, 1, 4 and 14 days in SBF are shown in Figure 9. The development of an apatitic layer on the surface of the samples was confirmed by SEM examination, which showed the presence of spherical particles with cauliflower morphologies, indicating the bioactivity of the bioglasses. The apatite particles agglomerated and became denser with prolonged immersion in SBF, leading to a completely covered surface by 14 d of immersion. These findings serve as compelling evidence of the potential osteoconductive properties of the prepared samples, highlighting their capacities to promote bone growth and regeneration. Furthermore, upon comparing bioglass samples containing varying Cu content, it was observed that the inclusion of high concentrations of Cu reduced the bioactivity of the material within the first day of immersion in SBF. This occurrence can be attributed to the conversion of bridging-oxygen ions (BOs) to NBOs, as presented in Figure 2d, leading to an increase in the glass connectivity, and therefore a drop in the dissolution rate and ions released.

**Figure 10:**
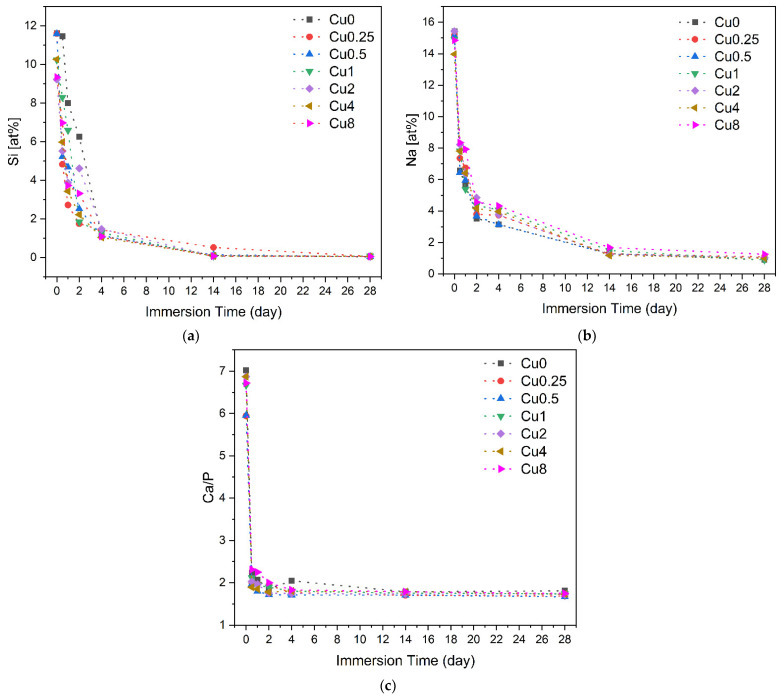
Variations in the concentrations of (**a**) Si; (**b**) Na; (**c**) Ca/P ratio on the bioglass pellets surfaces after immersion in SBF.

The reaction mechanism and ionic exchange between the bioglass and the SBF medium were also observed through SEM–EDS analysis. This analysis revealed the changes in atomic percentages of chemical elements on the sample surfaces as the immersion time in SBF increased. Figure 10 presents the variations in the atomic percentages of Si, Na and the Ca/P ratio with the immersion time on the surfaces of different bioglass samples. Upon immersion in SBF, the bioactive glass engages in an ionic exchange with the surrounding medium, leading to a fast release of soluble ionic species. Within the first 24 h, the soaking triggers the development of a surface layer of Si–OH, resulting in a rise in pH and subsequent formation of soluble Si(OH)_4_. This process leads to the creation of a silica gel layer, enabling the absorption of ions from the surrounding environment. Concurrently, Ca^2+^ and phosphate (PO_4_^3−^) diffuse through this layer towards the sample surface, promoting the formation of an amorphous calcium phosphate film. Over time, this amorphous CaP-rich layer undergoes crystallization [7,71]. Figure 10a,b illustrate a notable reduction in Si and Na concentrations on the sample surfaces during the initial days, followed by a subsequent stabilization in the succeeding days. This phenomenon is attributed to the dissolution of these elements into the surrounding medium, and the formation of a layer rich in Ca and P. In addition, the Ca/P confirms the formation of an apatite layer, revealing a value that is close to the Ca/P ratio of hydroxyapatite in natural bone (Ca/P ≈ 1.67) [72,73]. The Ca/P ratio for the Cu0.5 sample reached a value of 1.79 within the first day of immersion in SBF, compared to 2.07 for the Cu0 sample. This indicates a positive effect of copper on the bioactivity of the glass, promoting bioactivity within the initial 24 h, as evidenced by the faster approach of the Ca/P value towards those of hydroxyapatite.

**Figure 11:**
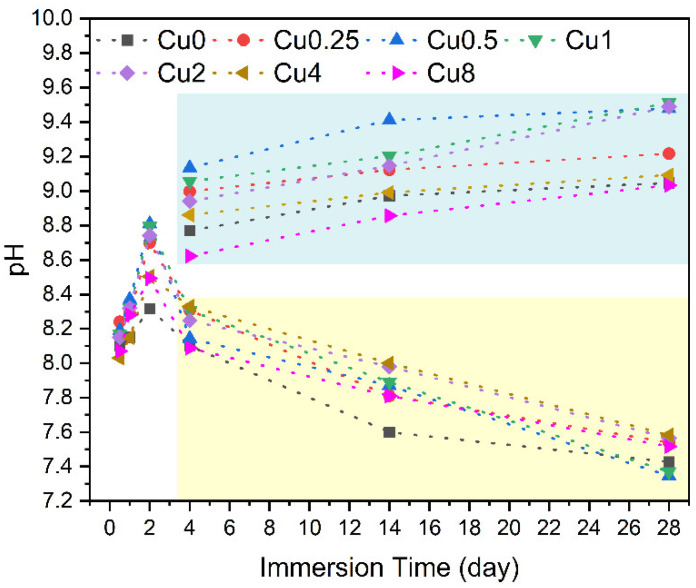
Variation in pH values of the SBF solution with the immersion times of all bioglass samples, with the medium changed every two days (yellow rectangle) and without medium change (blue rectangle).

The pH levels of the SBF were monitored at different intervals of time for all glasses, regardless of whether the medium was changed every two days or not. The obtained results are depicted in Figure 11.

As mentioned earlier, the pH of the SBF solution surrounding the bioglass exhibited a gradual increase throughout the immersion time due to the dissolution of alkaline metal ions (Na^+^ and Ca^2+^) from the samples. During the initial two days, this rise was particularly pronounced, and the increment became more gradual during the remaining period, as indicated by the values enclosed within the blue rectangle (corresponding to samples where the medium was not changed). Nevertheless, upon simulating the conditions resembling the in vivo environment by changing the medium every two days, the pH decrease was observed that is attributed to the formation of the apatite layer on the surface of the bioglass.

## 4. Conclusions

The synthesis of bioactive 45S5 glass samples was successfully achieved using the melt-quenching technique, incorporating varying amounts of copper oxide (CuO). Analysis using FTIR and Raman revealed no changes in the glass matrix upon CuO addition. The deconvolution of the Raman spectra demonstrated a decrease in the amount of Q1, Q2 and Q3 units with copper insertion, suggesting a decrease in NBOs concentration, and thus a reduction in the glass dissolution rate. Moreover, the PL spectra showed the presence of copper in the glass network in two oxidation states, Cu^+^ and Cu^2+^, which have an effect on the antibacterial properties of the bioglass. It was observed that the antibacterial properties decreased for the samples with a high content of CuO, where Cu^2+^ ions predominate over Cu^+^ ions. The antibacterial evaluation against *E. coli*, *S. aureus* and *S. mutans* indicated that the 0.5 mol% CuO-loaded glass demonstrated the most substantial antibacterial effect. The assessment of cytotoxicity for these glasses demonstrated that the incorporation of copper up to 4 mol% into the 45S5 bioglass did not induce adverse effects on a Saos-2 cell line at extract concentrations below 25 mg/mL. The in vitro immersion tests in SBF showed that the addition of low copper content enhances the bioactivity characteristic of 45S5 glass. This study offers valuable insights for the future advancement of antibacterial coatings for implants using copper-containing bioglass.

## Data Availability

The data presented in this study are available on request from the corresponding author.
